# Special considerations for women with hidradenitis suppurativa

**DOI:** 10.1016/j.ijwd.2020.02.005

**Published:** 2020-02-19

**Authors:** Erin Collier, Vivian Y. Shi, Ram K. Parvataneni, Michelle A. Lowes, Jennifer L. Hsiao

**Affiliations:** a David Geffen School of Medicine, University of California, Los Angeles, CA, United States; b Division of Dermatology, Department of Medicine, University of Arizona, Tucson, AZ, United States; c Department of Obstetrics and Gynecology, University of California, Los Angeles, CA, United States; d The Rockefeller University Laboratory for Investigative Dermatology, New York, NY, United States; e Division of Dermatology, Department of Medicine, David Geffen School of Medicine, University of California, Los Angeles, CA, United States

**Keywords:** Hidradenitis suppurativa, Inflammatory skin disease, Pregnancy, Lactation, Women’s health

## Abstract

Hidradenitis suppurativa (HS) is a chronic, debilitating disease that manifests as painful nodules, abscesses, sinus tracts, and scars with a predilection for intertriginous sites. HS disproportionately affects women of childbearing age and often leads to impairments in patients’ health-related quality of life. Women with HS face unique challenges related to menstruation, pregnancy, and lactation that require additional strategies for optimization of management. Practical interventions include lifestyle modifications, treatment of premenstrual HS flares, enhancing HS management during pregnancy, and creating optimal delivery plans in collaboration with obstetricians. This discussion is based on expert recommendations and aims to highlight the special challenges for women with HS, as well as provide a practical discourse on optimizing care of female patients with HS.

## Introduction

Hidradenitis suppurativa (HS) is a chronic, debilitating dermatosis that manifests as painful nodules, abscesses, sinus tracts, and scars in intertriginous sites (Alikhan et al., 2019). The reported average annual incidence in the United States is 12.1 per 100,000 women, which is more than twice that of men (5.1 per 100,000). Furthermore, nearly three quarters of new diagnoses are made in women. Women of childbearing age are disproportionately affected by HS, with the highest incidence of HS in patients aged 30 to 39 years (Garg et al., 2017). The pathophysiology of HS is unclear, but is likely a multifactorial process with genetic, environmental, and hormonally mediated factors, some of which can be modifiable (Smith et al., 2017).

Risk factors associated with HS aggravation included sweating, heat, exercise, stress, fatigue, tight clothing, weight, and menstrual periods (von der Werth and Williams, 2000). Given the clinical manifestations and exacerbating factors of HS, many patients face obstacles in performing activities of daily life. Women additionally face unique challenges related to menstruation, pregnancy, and breastfeeding. Herein, we propose strategies based on our expert recommendations to optimize care in female patients with HS and improve their quality of life (QoL; Table 1).

**Table 1:** Summary of practical considerations for optimizing care of HS disease in women.

Special consideration	Recommendations
Lifestyle modifications	• Implement weight loss plan via diet modification and exercise • Early referral for bariatric surgery consultation, if appropriate • Encourage identification of possible disease activity triggers using symptom and daily diary • Suggest low-intensity and low-impact exercise to minimize overheating and excessive perspiration • Design exercise regimens with minimal friction in areas of active HS lesions • Consider calcium and vitamin D supplementation • Advise wearing loose-fitting, breathable clothes with 100% cotton, rayon, or bamboo fibers • Recommend sports bras or camisoles with built-in wireless bras and women’s briefs or boy shorts to decrease friction • Recommend moisture-wicking fabric that can be cut to fit affected intertriginous regions • Discuss trying gentle deodorizing antiperspirant sprays • Offer laser hair removal to improve symptoms and try to prevent disease progression • Advise against shaving in areas of active lesions • Provide resources for women’s smoking cessation programs (e.g., women.smokefree.gov)
Menstruation considerations (in women not considering pregnancy)	• Consider estrogen and antiandrogenic progesterone-containing oral contraceptives in patients prone to premenstrual HS flares • Consider spironolactone in conjunction with oral contraceptives in women with childbearing potential • Counsel patients with groin lesions to use tampons during menses
Pregnancy considerations	• Counsel patients on appropriate weight gain during pregnancy • Discuss optimal method of delivery with obstetrics–gynecology specialists when needed; severe genital HS lesions may influence choice of delivery method or cesarean section scar placement • Recommend patients with gestational diabetes consider consultation with maternal–fetal medicine for evaluation of need for insulin • Recommend close monitoring of patients for anemia • Discuss supplemental oral zinc • For patients with severe HS, may discuss use of biologic agent throughout pregnancy • Avoid live vaccinations for first 6 months in babies born to mothers who were on biologics throughout pregnancy
Breastfeeding considerations	• Counsel patients that inframammary lesions should not affect baby’s ability to latch and feed • Treat HS breast lesions early, prior to delivery, to facilitate breastfeeding • Inform patients that breastfeeding while on anti–tumor necrosis factor-alpha antagonists is generally considered safe

HS, hidradenitis suppurativa.

## Practical interventions

### Lifestyle modifications

Patients should be counseled on strategic lifestyle modifications to mitigate HS exacerbations (Alikhan et al., 2019). Obesity is a major comorbidity of HS and is hypothesized to play a role in lesion development, likely by predisposing to a systemic inflammatory state, mechanical friction from enlarged breasts and skin folds, and a humid environment favoring skin microbial growth (Kromann et al., 2014a). Women experience barriers to engaging in physical activity, including lack of time, lack of enjoyment, self-consciousness, urinary incontinence, and lack of child care (Moreno and Johnston, 2014). Thus, encouraging weight loss via dietary modifications and achievable exercise is critical, although this well-intentioned advice may be better received after a therapeutic alliance has already been established.

Early referral to bariatric surgery evaluation in morbidly obese patients with HS should be considered. Patients who have achieved significant weight loss can experience HS flares in their loose panniculus and may require excision of excess skin (Sivanand et al., 2020). Patients who undergo bariatric surgeries involving intestinal bypass should be closely monitored for malabsorption and nutritional deficiencies (Garcovich et al., 2019).

### Exercise

Low-intensity and low-impact exercises that minimize overheating, such as Pilates, yoga, and swimming, should be encouraged. Wearing loose clothing and applying a barrier cream or sweat-absorbing powder prior to exercise can decrease skin friction. Alternatively, an exercise regimen tailored to specific anatomic areas can help avoid regions of flare. For example, for women with menstrual flares of groin HS, upper body–focused exercises can be recommended. For women facing barriers to exercise, a discussion of lifestyle activities, such as taking the stairs, playing outdoors with children, and walking instead of driving, can be helpful (Moreno and Johnston, 2014).

### Diet

Elimination of dairy and brewer’s yeast have reported benefits in HS (Sivanand et al., 2020). Given the higher rates of osteoporosis in women than in men, if dairy intake is limited, patients should be encouraged to select fortified foods or supplements with calcium and vitamin D (Guillet et al., 2015). Furthermore, following a Mediterranean-style diet that is high in antioxidants and polyphenols decreases HS severity through anti-inflammatory effects (Barrea et al., 2019). A symptom and daily diary may enable patients to identify possible food triggers of disease activity. Referral to a nutritionist to assist with formulating a dietary plan can be beneficial.

### Clothing

Friction from tight clothing can stimulate epidermal hyperplasia and thickening to exacerbate HS (Alikhan et al., 2019). Provide specific clothing recommendations that minimize friction and irritation, especially in intertriginous areas affected by HS. Patients should be encouraged to wear loose-fitting and breathable clothing. Fabric with 100% cotton, cellulose-derived rayon, or bamboo fibers are soft and absorbent. Sports bras and camisoles with built-in wireless bras can help offload pressure and friction from inframammary areas. Additionally, boy shorts and high-cut briefs may decrease subpannus friction in women (Loh et al., 2019).

### Wound care and dressings

Wound care helps control drainage and associated staining of clothing (Alavi et al., 2018). Biker shorts are useful for holding groin dressings in place. Tubular elastic bandages can be cut to support axillary dressings. Moisture-wicking fabric with silver can be cut to size to fit in inframammary, axillary, and groin regions. Cool air from a hair dryer can be used to gently dry skin folds after bathing. For women who experience malodor, gentle deodorizing antiperspirant sprays can be tried after first testing on a nonsensitive area.

Patients with draining vulvar, perineal, or inferior buttock HS lesions may be counseled to wear adult diapers or soft hygiene overnight-size pads as a form of wound care. In times of heavy drainage, patients may also wear disposable postpartum mesh underwear as a breathable outer fabric that comfortably holds pads in place.

### Shaving and hair reduction

In general, patients should be counseled to avoid shaving of the axilla and pubic region when active lesions are present to avoid worsening of lesions. Laser hair reduction can improve HS lesions, help prevent future eruptions, and help women achieve hairless skin if they desire it (Tierney et al., 2009). Of note, in a small study, local microwave ablation treatment was not beneficial (Vossen et al., 2019).

### Tobacco exposure

Smoking status is correlated with HS disease severity (Alikhan et al., 2019). Tobacco exposure has been shown to decrease activity of the Notch transmembrane signaling pathway that may be involved in HS pathogenesis (Smith et al., 2017). Consider directing patients to women-specific smoking cessation programs with cognitive behavioral strategies for managing triggers of cravings (e.g., women.smokefree.gov). Valuable components of these programs include addressing fears of weight gain and body image as a reason for possible relapse and managing the interpersonal stress of balancing family and work (Torchalla et al., 2012).

### Menstruation

Women often note premenstrual HS exacerbation, with as much as 43% of women reporting worsening of disease around menses (Vossen et al., 2017). Premenstrual decrease in progesterone levels may be a contributing factor because progesterone suppresses CD4+ T cell proliferation and inhibits Th17 T cell differentiation (Perng et al., 2017a). Thus, patients who do not desire pregnancy may benefit from estrogen and antiandrogenic-progesterone-containing oral contraceptive pills. Spironolactone can be added for its antiandrogenic properties (Golbari et al., 2019). Tampons can minimize discomfort and friction with groin lesions during menstruation (Margesson and Danby, 2014).

### Pregnancy and delivery

The effects of pregnancy on HS are mixed (Kromann et al., 2014b). An early discussion of potential worsening of HS during pregnancy and available treatment options can provide the patient with anticipatory insight. If a woman is on a biologic agent, consider discussing continued administration during pregnancy with her obstetrician to mitigate disease flaring. Tumor necrosis factor-alpha antagonists are generally considered safe and have the most pregnancy-related data for the first half of pregnancy (Porter et al., 2017). Recommendations can be made for longer-term use depending on HS severity. Patients on biologic treatment throughout pregnancy should be counseled to avoid administration of live vaccinations (e.g., rotavirus vaccine) to their babies in the first 6 months of life due to an increased risk of infection (Porter et al., 2017).

If gestational diabetes develops, consider referral to maternal–fetal medicine for evaluation of the need for insulin. Alternative treatment options such as oral zinc 90 mg/day can also be tried to suppress HS flares during pregnancy due to its anti-inflammatory properties and favorable safety profile (Brocard et al., 2007).

Patients with HS have been found to have a higher incidence of anemia compared with controls (Soliman et al., 2020), and pregnancy is also associated with anemia. Thus, pregnant patients with HS should be monitored carefully for anemia because anemia during pregnancy is a risk factor for low birth weight and preterm delivery (Allen, 2000).

Patients should also be counseled on appropriate weight gain during pregnancy (American College of Obstetricians and Gynecologists, 2013). The abdominal pannus is an area where HS severity may worsen during pregnancy. Weight gain further contributes to HS pathogenesis through increasing mechanical stress in intertriginous areas, which leads to retention of hair follicle materials, epidermal hyperplasia, and follicular rupture (Boer et al., 2016).

In women with active vulvar HS lesions, collaborating with obstetrics–gynecology to discuss delivery can reduce the risk of HS exacerbation and intrapartum tissue damage. From the authors’ experiences and discussions with obstetrics–gynecology colleagues, we find that a cesarean section may be more appropriate for women with severe vulvar disease. Additionally, some women who require a cesarean section delivery may have severe or secondarily infected HS in the lower abdomen. In this case, consider having a discussion with obstetrics–gynecology specialists regarding the feasibility of changing the surgical incision to avoid the area of active HS.

### Breastfeeding

Patients should be informed that inframammary HS lesions do not usually affect their baby’s ability to latch and feed. For patients with HS lesions on their breasts who plan to breastfeed, early treatment of lesions with intralesional corticosteroids, oral antibiotics, or other medications prior to delivery can make breastfeeding more feasible. Patients can also be counseled that breastfeeding while on an anti–tumor necrosis factor-alpha antagonist is generally considered safe due to minimal amounts passing into breast milk (Perng et al., 2017b).

## Conclusion

HS is a debilitating disease that disproportionately affects women of childbearing age. Women with HS deserve a comprehensive approach to disease management with special considerations during menstruation, pregnancy, and lactation. These practical recommendations allow providers to tailor strategies to optimize care of their female patients with HS. Further investigation into the unique challenges faced by women with HS and outcomes of women-specific HS management strategies is needed. Prospective studies (Adelekun et al., 2020) are underway to collect data on HS during pregnancy and guide future recommendations.

## Conflict of Interest

None

## Funding

The authors made the following disclosures: V.Y. Shi is a stock shareholder of Learn Health and has served as an advisor, investigator, and/or speaker for Sanofi Genzyme, Regeneron, AbbVie, Burt’s Bees, Dermira, Eli Lilly, Novartis, Pfizer, Galderma, Leo Pharma, SUN Pharma, Menlo Therapeutics, GpSkin, and Skin Actives Scientific. There was no financial transaction for the preparation of this manuscript. M.A. Lowes has served on the advisory board for AbbVie and Janssen and as a consultant for AbbVie, XBiotech, BSN, Almirall, and Incyte, unrelated to this manuscript. R.K. Parvataneni, E. Collier and J.L. Hsiao, report no conflicts of interest.

## Study Approval

None.
